# Effect of different instrumental techniques and clinical experience on shade matching

**DOI:** 10.1111/jopr.13894

**Published:** 2024-06-13

**Authors:** Sina Saygılı, Berkman Albayrak, Tonguç Sülün

**Affiliations:** ^1^ Department of Prosthodontics Istanbul University Faculty of Dentistry Istanbul Turkey; ^2^ Department of Prosthodontics Bahçeşehir University School of Dental Medicine Istanbul Turkey

**Keywords:** color analysis, cross‐polarization, digital photography, intraoral scanner, shade matching

## Abstract

**Purpose:**

Many factors can affect the aesthetics of dental restorations, including the instrumental techniques used in shade matching, and can lead to clinical failure. The aim of this study was to investigate the effectiveness of using the cross‐polarization digital photograph technique and intraoral scanners for shade matching, and also evaluate the effect of the level of clinical experience on shade matching success.

**Materials and Methods:**

Color analysis was performed on the maxillary right central incisors of 10 subject models with Vita Easyshade. Intraoral scanning was performed 10 times on each model using TRIOS 3 and color analysis was performed from the same spot. Then cross‐polarized and non‐polarized photographs of the models were taken with standard settings using a gray reference card. Each shade tab of the Vita System 3D‐Master scale was also photographed with two different polarization techniques. Four groups (*n* = 12), including prosthodontics faculty staff, postgraduate students in prosthodontics, undergraduate students, and dental technicians matched the shade tabs and the model photographs obtained with both techniques on a standardized computer screen. Finally, the color differences between the shade tabs and maxillary central incisors matched by observers from four different groups were recorded using a colorimeter, Classic Color Meter, in accordance with the CIELAB system and CIEDE2000 (ΔE_00_) values were calculated. The data were compared with the acceptability threshold of 1.80 for ΔE_00_. The data obtained from the observers were analyzed with IBM SPSS Statistics 26.0 Release Notes program. Independent Samples *t*‐test was used to compare normally distributed data according to binary groups. The level of significance was 0.05.

**Results:**

A statistically significant difference was found in the shade matching on photographs taken with different techniques in postgraduate students (*p* = 0.02). Also, there was a statistically significant difference in success between the groups that made shade matching based on photographs obtained with the non‐polarization technique (*p* = 0.00). The undergraduate students achieved statistically significantly lower results than all other groups (ΔE_00_ = 5.57 ± 1.07). The kappa value between the intraoral scanner and spectrophotometer results was 0.10, and this value was not statistically significant (*p* = 0.32).

**Conclusions:**

The cross‐polarization technique used especially for shade matching is not superior to the non‐polarization technique. Academic and clinical experience might be correlated with shade‐matching success with the non‐polarization technique. The clinical acceptability threshold could not be achieved in the shade matchings made on digital photographs taken with both techniques. Shade matching performed with an intraoral scanner did not yield reliable results.

One of the most important success criteria in aesthetic restorative dentistry is to ensure the color and shape harmony between the applied restoration and the remaining teeth or existing restorations.^1^ The success at this stage depends on three main factors such as the correct determination of the current color of the remaining dentition, the delivery of this color and shape to the laboratory with an accurate data transfer method, and the production of the ceramic in such a way as to achieve this harmony.^2^, ^3^, ^4^ Although visual and instrumental methods are used in clinical shade matching, which is the first step of these procedures,^5^ a standardized protocol has not been developed.^6^

In shade matching, Munsell, which includes hue, value, chroma parameters, and CIELAB color systems that can present numerical data with L* a* b* parameters, is used.^7^ The CIELAB  color system is used to define a specific color with parameters L* related to value, a* with the color scale from red to green, and b* with the color scale from yellow to blue.^7^ ΔE*_ab_ and more recently introduced CIEDE2000 (ΔE_00_) are used to measure the color difference, and to assess the level of perception and acceptability of the difference between two colors.^8^, ^9^ Thanks to ΔE_00_, the success of the restorations in terms of color harmony can be evaluated. Paravina et al.^10^ reported that the acceptability thresholds are designated as excellent match for ≤0.8, acceptable match for ≤1.8, moderately unacceptable for ≤3.6, clearly unacceptable for ≤5.4, and extremely unacceptable for >5.4.

Clinical shade matchings are carried out by two different methods, visual and instrumental. In visual shade matching, Vita System 3D‐Master (Vita Zahnfabrik, Bad Säckingen, Germany), which prioritizes value in visual shade matching, and Vita Classical A1‐D4 (Vita Zahnfabrik, Bad Säckingen, Germany) and Chromascop (Ivoclar Vivadent, AG, Schaan, Lietschentein) guides, which have hue parameter priority, are used.^11^ However, in shade matchings performed with these guides, various failures may be encountered due to inconsistencies between the shade tabs produced by the same manufacturer,^12^ inadequacies in the variation of shade tabs,^13^, ^14^ and the lack of color standardization in ceramic products produced by different manufacturers.^15^ In addition, it has been reported that the age, experience, dental education level, eye fatigue, color blindness of the operator performing the shade matching, and light exposure level also affect the success.^16^, ^17^, ^18^, ^19^, ^20^, ^21^, ^22^, ^23^

The instrumental method aims to make more objective and reproducible shade matching by using spectrophotometers, colorimeters, intraoral scanners, and digital cameras.^24^, ^25^ According to research, more stable results are obtained with spectrophotometers and colorimeters in shade matching.^26^ Gehrke et al.^27^ and Dozic et al.^28^ reported that the most reliable results are obtained with spectrophotometers. Among the spectrophotometers, Vita Easyshade (Vita Zahnfabrik, Bad Säckingen, Germany) reveals the best quality data in terms of accuracy and precision.^7^, ^28^

Digital photography, on the other hand, constitutes an important alternative among instrumental methods in terms of determining the color and transferring the data in the mouth to the technician more easily.^29^ Thus, polychromatic colors in the mouth, and properties such as morphology, surface texture, and translucency of the remaining teeth and restorations can be transferred accurately.^1^, ^30^ For shade matching, the cross‐polarization technique is recommended in order to eliminate the brightness of the room where the photograph was taken and the reflection of the flash on the image caused by saliva, tooth structure, and restorations.^31^, ^32^ In the cross‐polarization technique, standard gray reference cards (white balance, Emulation) produced with L:79 a:0 b:0 are located next to the teeth whose color determination is to be made. Afterward, the exposure balance of the dental photographs is performed in graphic software such as Adobe Photoshop until the measured luminosity of the gray card matches L*79.  Thus the effect of value on the surface can be eliminated, higher contrast images can be obtained and more objective shade matchings can be achieved.^33^ The cross‐polarization technique makes an important contribution to color determination as it reduces the glare created by the flash on the tooth by 30%.^34^ Although promising results have been obtained in studies carried out with the cross‐polarization technique,^35^, ^36^ some studies have reported that the value of the tooth to be shade matched may affect the success of this technique.^37^

Current intraoral scanners also have color‐determination functions. Devices such as TRIOS 3 (3Shape Trios A/S, Copenhagen, Denmark) allow color determination from any spot in accordance with both the Vita System 3D‐Master and Vita Classical A1‐D4 scales. However, there are studies showing that color determinations performed with intraoral scanners provide better results than visual techniques,^38^, ^39^ and also studies showing that they are not reliable.^40^, ^41^ In clinical practice, intraoral scanners still have not found widespread use for shade matching.

The aims of this study are to evaluate the effect of the cross‐polarization technique used in digital photographs, the effect of clinical experience on shade matching, and lastly the color analysis success of intraoral scanners. The first hypothesis of the study is that the cross‐polarization technique has no effect on shade matching. The second hypothesis is that there is no difference in the success of groups with different clinical experience levels and the third hypothesis is that intraoral scanners successfully determine color.

## MATERIAL AND METHODS

The study protocol was approved by the Istanbul University Faculty of Dentistry, Clinical Research Ethics Committee (decision number: 424, date: 20.06.2019). The sample number of the study was calculated with G*Power Version 3.1.9.2. Calculations determined that it was appropriate to work with 12 people in four different groups, based on the correct match rates of the research conducted by Schropp et al.^1^ (based on the computer software 67% and the photograph 28%), one‐way alternative hypothesis, 80% power, and 5% Type I error margin. Ten undergraduate dentistry students between 22 and 25 years old were selected, and consent documents were obtained for participation after the study concept was explained. The inclusion criteria were determined as the absence of tooth loss in the anterior region, the absence of discoloration of the anterior teeth due to any restoration, root canal treatment, or any other reason. Students who had bleaching treatment in the last 6 months and insufficient oral hygiene were excluded.

**FIGURE 1:**
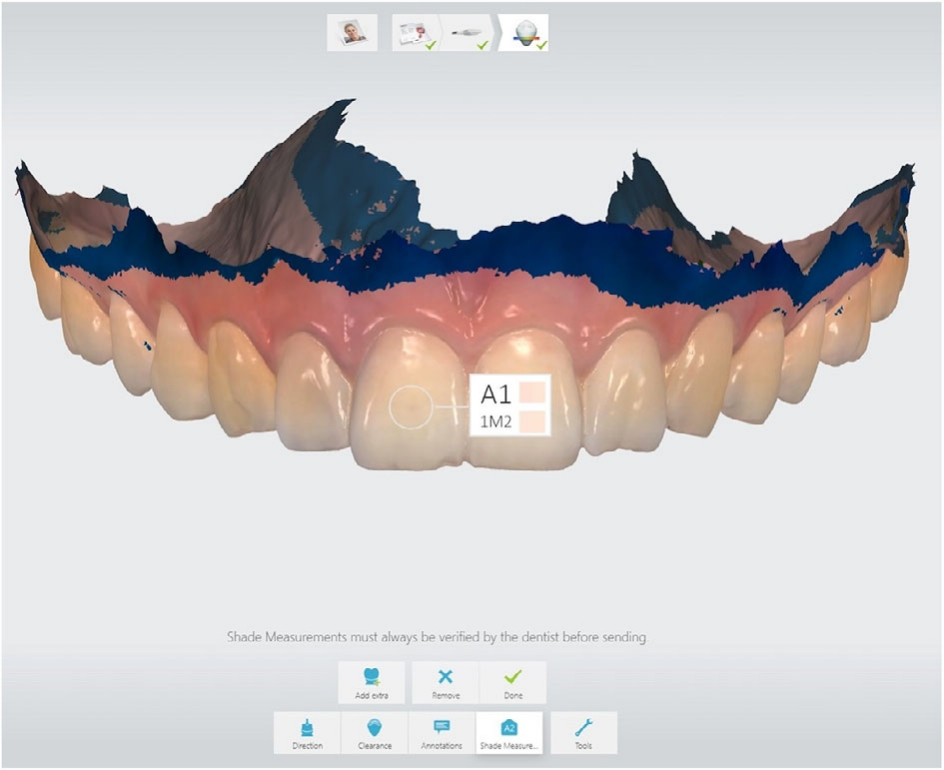
Shade matching on intraoral scan.

Afterward, all subject students were informed about the shade matching with the instrumental technique and photographs, and before the procedures, they were asked to brush their teeth and not to use makeup materials such as lipstick, etc. First, color measurements were performed with a spectrophotometer (Vita Easyshade Advance 4.0; Vita Zahnfabrik), which is accepted as the gold standard, by taking the center of the maxillary right central incisors of the subject students as a reference. Then, digital impressions were made from all participants with the TRIOS 3 intraoral scanner (3Shape Trios A/S, Copenhagen, Denmark) 10 times. The TRIOS 3 intraoral scanner was calibrated for the color measurements according to the manufacturer's instructions before each measurement. The color measurements were made on the center of the maxillary right central tooth using the scanner software (3Shape Dental Software), according to the Vita System 3D‐Master scale and these values were recorded (Figure 1). All instrumental shade matching, digital impression, and color measurement stages were carried out by a single experienced operator (S.S.).

Ambient light between 5500 and 6500 K was used in the photography room. For standardized photography, subjects kept their heads upright and did not rotate, and the maxillary arch teeth were positioned parallel to the ground. By applying a retractor, the lips and cheeks were removed before taking the photographs. The mouths of the participants were closed in order to prevent dehydration between photographs to be taken with different techniques. With the help of a tripod, a DSLR camera (Nikon D7500), a 105 mm macro lens (Nikon), and twin flash (Nikon R1C1) photographic setup was created and fixed. Camera/flash settings; manual flash, AF aperture F22, shutter speed 1/125 s, ISO 200, magnification 1:2.5, and White Balance adjusted to 5500 K. Before photographing, the maxillary central tooth‐lens distance was set to 45 cm.

**FIGURE 2:**
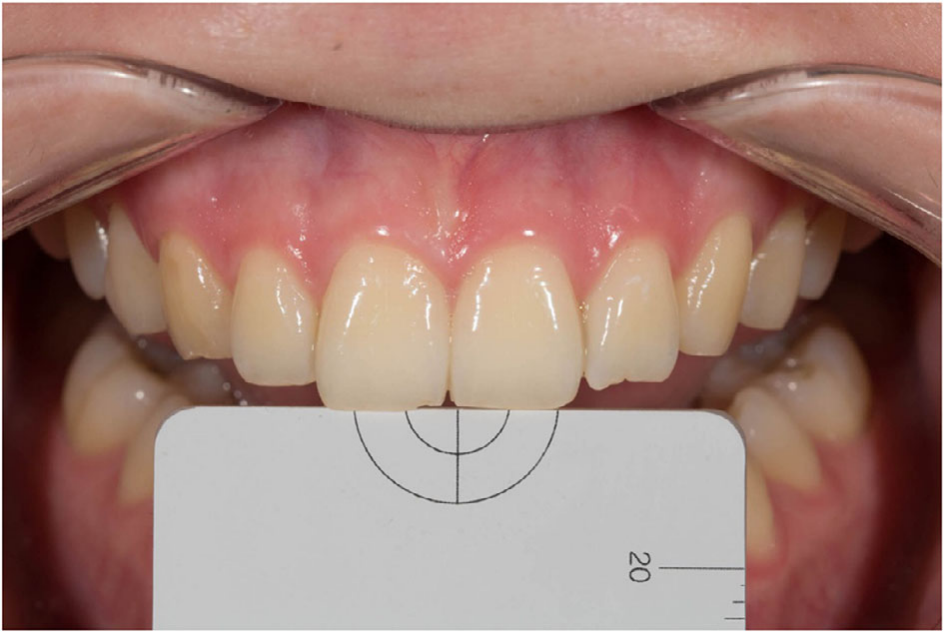
Non‐polarized digital dental photograph.

**FIGURE 3:**
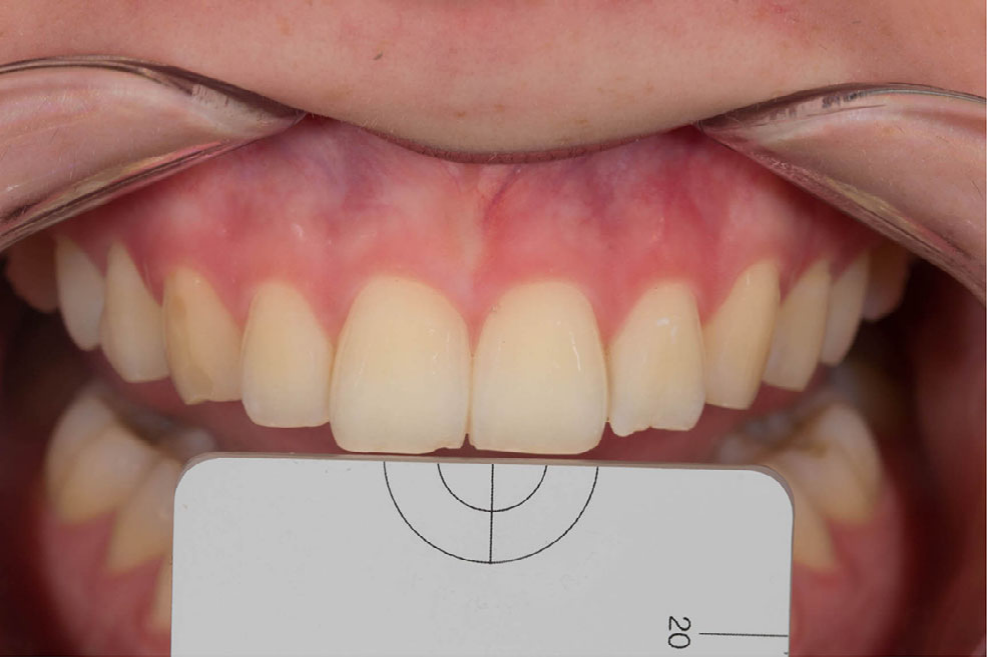
Cross‐polarized digital dental photograph.

**FIGURE 4:**
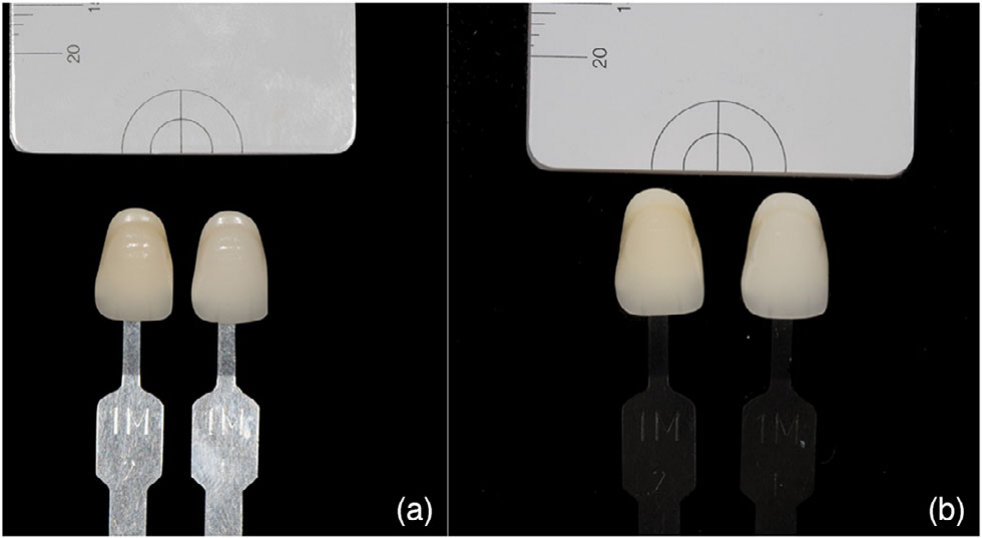
Digital dental photographs of shade tabs: (a) Non‐polarized photograph. (b) Cross‐polarized photograph.

First, non‐polarized photographs were taken from the region between the maxillary right and left canines of each participant (Figure 2), then cross‐polarized photographs were taken by placing standard gray reference cards (white balance, Emulation) produced with L:79 a:0 b:0 optical properties on the coronal part of the teeth (Figure 3). All photographs were taken by using Crop Sensors APS‐C (DX) and cross‐polarized photos were obtained in RAW format, and they were edited with the values of L:79 a:0 b:0 in a graphic software (Lightroom v6.0, Adobe Photoshop CC; Adobe Systems Inc.) with the help of reference gray cards. Then, each of the shade tabs in the Vita System 3D‐Master scale (Vita Zahnfabrik, Bad Säckingen, Germany) was photographed with the cross‐polarization technique with the help of the same standard gray reference card on a matte, black background. Then the non‐polarized photographs were also taken (Figure 4a,b). Exposure compensation of cross‐polarized photographs was performed with the same values in Adobe Photoshop Lightroom and saved.

**FIGURE 5:**
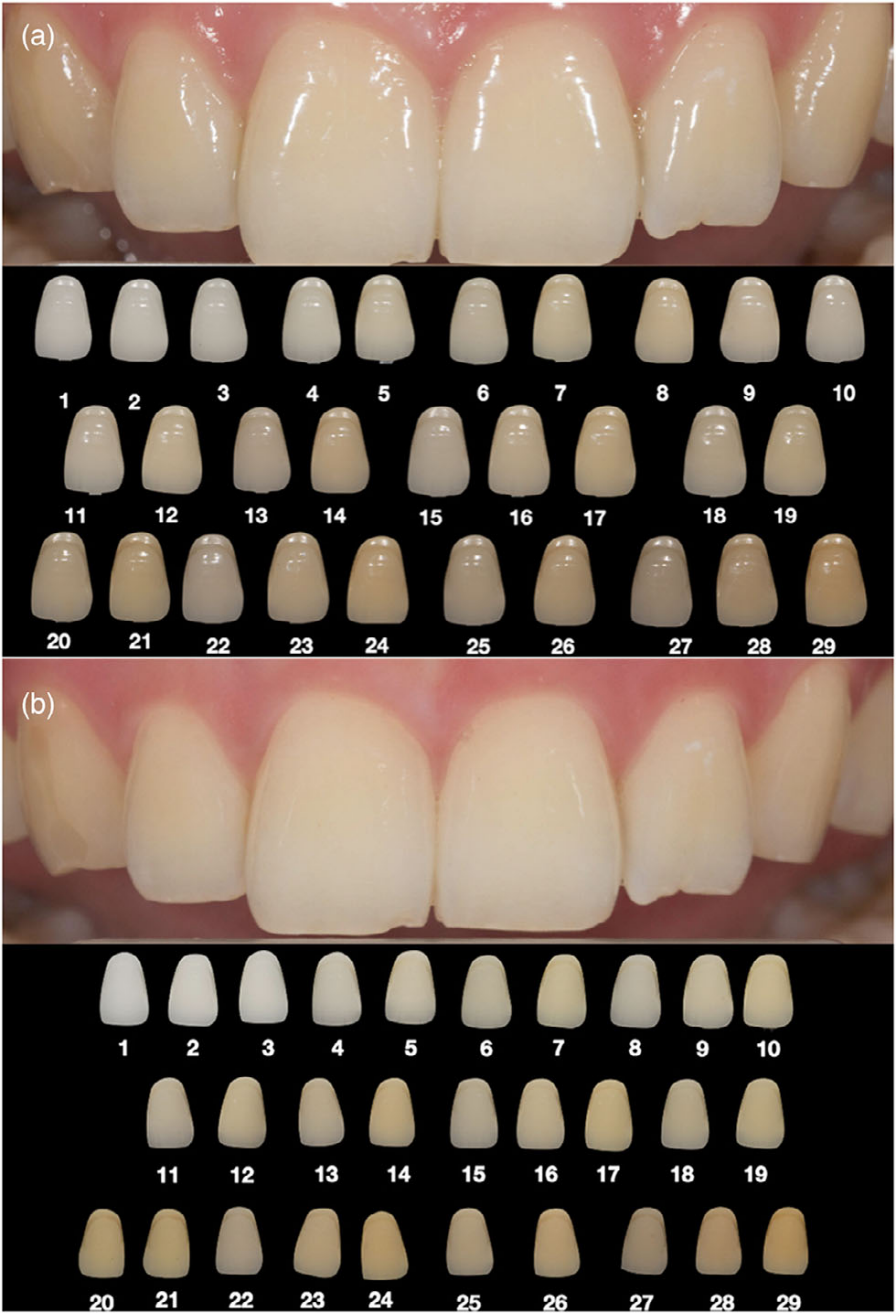
Presentation of the subject photographs and shade tabs on the screen. (a) Non‐polarized photographs. (b) Cross‐polarized photographs.

Color measurements were performed with a digital colorimeter software (Classic Color Meter version 5.22 for MacIntosh AC; Ricci Adams) with reference to the center of the right maxillary central teeth on non‐polarized and cross‐polarized digital photographs of the subjects and shade tabs, and CIELAB values were recorded. Then, the photographs were loaded into a presentation program (Keynote version 5.22 for MacIntosh AC; Ricci Adams) to match the shade tabs photographed with the same technique as non‐polarized and cross‐polarized subject photographs. A non‐polarized subject photograph and all non‐polarized shade tabs were added to each slide in a randomized manner, and then a cross‐polarized subject photograph and cross‐polarized all shade tab photographs were added to the next slide (Figure 5a,b).

A total of 48 observers from four different academic groups with different levels of experience were selected, including faculty staff working in the Department of Prosthodontics of Istanbul University Faculty of Dentistry, postgraduate students doing their doctorate in the same department, undergraduate intern students who had completed their internship in prosthodontics, and dental technicians (n: 12). Observers successfully passed the color blindness test and were included in the study. These observers performed shade matching with the shade tabs by referring to the center of the maxillary right central tooth on the digital photographs placed in the Keynote presentation program opened on the Macbook Pro Retina (Apple Company) screen with the same screen brightness and quality. The answer given by each observer was recorded, and the L* a* b* values ​​of the matched subject's tooth shade and shade tab, which were previously determined with the help of a colorimeter, were also recorded. In order to determine the difference between the shade choices made by the observers and the actual maxillary central tooth values ​​of the subject students obtained with Vita Easyshade, the CIEDE2000 color difference (ΔE_00_) was calculated as follows:^42^

$$\Delta E_{00}=\sqrt{\left(\frac{\Delta L^{\prime }}{kLSL}\right)2+\left(\frac{\Delta C^{\prime }}{kCSC}\right)2+\left(\frac{\Delta H^{\prime }}{kHSH}\right)2+RT\left(\frac{\Delta C^{\prime }}{kCSC}\right)\left(\frac{\Delta H^{\prime }}{kHSH}\right)}$$

Data were analyzed with IBM SPSS Statistics 26.0 Release Notes program (IBM Corp., Armonk, NY, USA). The suitability of the data for normal distribution was examined with the Shapiro‐Wilk test. Independent Samples *t*‐test was used to compare normally distributed data between two groups. Repeated Measures Analysis was used to compare the ΔE_00_ value, which was normally distributed according to observer groups, and multiple comparisons were examined with the Bonferroni test. Intraclass Correlation coefficient was used to examine the agreement of the ΔE_00_ parameter between and within observers. The compatibility of the intraoral scanner and the spectrophotometer was examined with Kappa. 0.05 was used as the level of significance.

## RESULTS

Forty‐eight observers performed shade matching on a total of 20 photographs taken with non‐polarized and cross‐polarized techniques from 10 student participants. A very good, statistically significant agreement was obtained between all study groups in ΔE_00_ parameter (Table 1).

**TABLE 1:** Examining the interobserver agreement of the ΔE_00_ value.

	ICC (%95 CI)	*p*‐Value
Dental Technicians	0.87 (0.77–0.94)	**<0.001**
Postgraduate Students	0.90 (0.82–0.95)	**<0.001**
Undergraduate Students	0. 85 (0.73–0.93)	**<0.001**
Faculty Staff	0.85 (0.73–0.93)	**<0.001**

Abbreviations: CI, confidence interval; ICC, intraclass correlation.

According to the shade matching performed on non‐polarized photographs, the three groups achieved statistically significantly better results than undergraduate students (*p* = 0.00). However, there was no statistically significant difference between postgraduate students, dental technicians, and faculty staff. In the shade matching made on cross‐polarized photographs, although the success ranking was dental technicians, faculty staff, postgraduate students, and undergraduate students, no statistically significant difference was obtained between the groups (Table 2).

**TABLE 2:** ΔE_00_ values of different academic groups matching shades in two different techniques.

	Technique			
Groups	Non‐polarized	Cross‐polarized	Test statistics	*p*‐value^d^
Dental Technicians	4.67 ± 0.76^a^	5.41 ± 0.98	−1.90	0.07
	4.72 (3.57–5.83)	5.40 (3.97–6.69)		
Postgraduate Students	4.43 ± 0.88^a^	5.40 ± 0.76	–2.63	**0.02**
	4.38 (3.15–5.62)	5.56 (4.07–6.58)		
Undergraduate Students	5.57 ± 1.07^b^	5.44 ± 0.98	0.29	0.77
	5.59 (3.88–7.22)	5.57 (3.98–6.60)		
Faculty Staff	4.86 ± 0.82^a^	5.48 ± 0.80	–1.74	0.10
	4.9 (3.39–5.69)	5.43 (4.27–6.49)		
Test statistics	11.78	0.13		
***p*‐value** ^c^	**0.00**	0.94		

^a‐b^ There is no difference between groups with the same letter.

^c^ Repeated Measurement Analysis.

^d^ Independent Samples *t*‐test.

**FIGURE 6:**
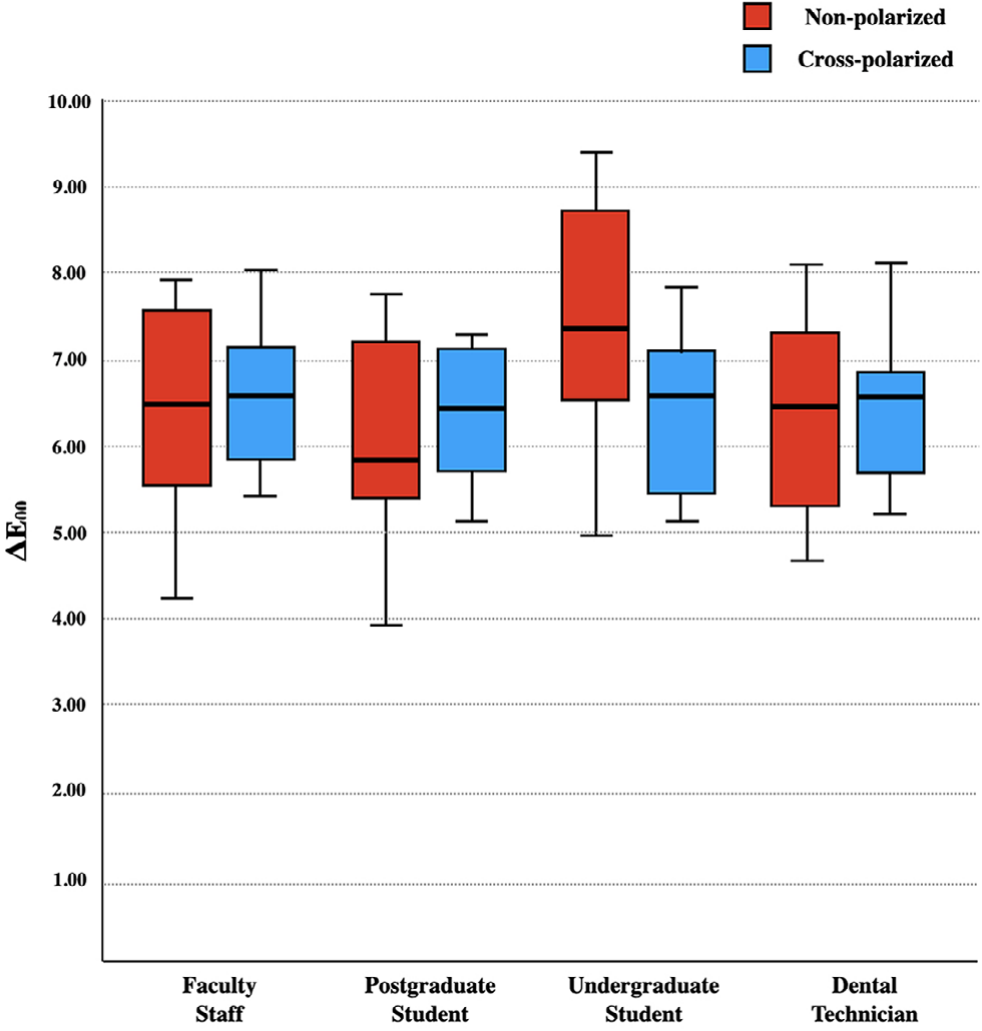
ΔE_00_ values of different academic groups in two different photograph techniques.

When the success of each group in non‐polarized and cross‐polarized photographs was evaluated, although all groups except the undergraduate students achieved better results in the non‐polarized technique, a statistically significant difference was detected only in the postgraduate students (*p* = 0.02). The ΔE_00_ value for postgraduate students was 4.43 ± 0.88 in the non‐polarized technique; a statistically significant unsuccessful result was obtained with 5.4 ± 0.76 in the cross‐polarized technique (Figure 6).

Finally, the color of the Vita System 3D‐Master scale obtained with a spectrophotometer from the exact center point of the maxillary right central teeth of the models was compared with the colors specified by the software from the same point in the intraoral scans. The kappa value (κ) between TRIOS 3 and Vita Easyshade was 0.10 and was not statistically significant (*p* = 0.32). Intraoral scanner and spectrophotometer data were found to be poor in terms of strength of agreement.

## DISCUSSION

Although there were no statistically significant differences in the three groups, the fact that the non‐polarization technique showed statistically significantly superior results for postgraduate students led to a partial rejection of the first hypothesis that cross‐polarized and non‐polarized photograph techniques would not affect shade‐matching success. The cross‐polarization technique, which was developed to prevent extreme light exposure that may occur on the tooth surface and to improve shade matching in dental photography, provided more successful results only in undergraduate students in the present study, but that success could not be achieved in other groups.

The second hypothesis that there would be no difference between the success of different academic groups in shade matching was also partially rejected. Although there was no difference between the shade matchings of the groups in the cross‐polarization technique, the undergraduate students were found to be statistically unsuccessful in the non‐polarization technique compared to the other groups. Along with factors such as age, eye disorders, and psychosocial status, an operator's shade‐matching experience can also affect success.^43^ The success of faculty staff who have experienced shade matching many times in prosthetic treatment processes has been determined to be lower than dental technicians and postgraduate students who have relatively less clinical experience.

The third hypothesis that intraoral scanners would be successful in color determination was completely rejected. Since the kappa value was below 0.20, it is considered poor in terms of strength of agreement. Therefore, intraoral scanners are required to be developed to be used for shade matching in clinical practice. In the present study, color determination of the intraoral scanner was carried out by using the Vita System 3D‐Master scale. However, Rutkunas et al.^40^ reported that color analyses performed by intraoral scanners with different scales can give various results.

As visual color selection is subjective, operator‐dependent, and highly influenced by existing light sources, instrumental shade‐matching techniques appear to be more reliable.^44^ Although the usage area of spectrophotometers and colorimeters developed for this purpose is expanding, these devices are designed to perform shade matching on flat surfaces. They have certain limitations on teeth with more complicated surfaces.^45^ Digital photographs can be taken with cross‐polarized filters and standard reference gray cards to eliminate the negative effect of light sources in shade matching.^31^, ^46^ The surface anatomy, translucency, soft tissue, and surrounding tissue structure of the teeth are better recorded with the photograph and more permanent references can be obtained for the production of a new restoration.^47^ However, the high costs of these color measuring devices and photography equipment may also limit their clinical use. They are not sufficient to reflect the characteristic features and localized discolorations of the tooth, as in digital photographs. In color analysis performed on digital photographs, the screen features and colorimeter software on which the photograph is evaluated may also affect the result. Although a standard screen and software were used in the present study, these parameters may affect the result differently than the visual shade‐matching technique.

The clinical acceptability of color difference is evaluated by comparing obtained ΔE_00_ values with the acceptability thresholds. Although there are different ΔE_00_ values accepted in many color studies, the acceptability thresholds were as excellent match for ≤0.8, acceptable match for ≤1.8, moderately unacceptable for ≤3.6, clearly unacceptable for ≤5.4 and extremely unacceptable for >5.4 according to Paravina et al.^10^ In the present study, higher ΔE_00_ values than extremely unacceptable values were obtained in shade matchings that were made on the shade tabs and human teeth. This reveals that performing shade matching with digital photography may not be sufficient to achieve clinically successful results. However, there is also a color difference between the human teeth and the shade tabs.^48^ It should also be taken into account that this situation may have affected the results obtained from the shade matching made on digital photographs in the present study.

Studies regarding the effect of clinical experience and training on shade matching show different results. In a study on undergraduate students conducted by Clary et al.^49^  , shade matchings were made on shade tabs, then the students were subjected to a lesson on color and repeated their shade matchings. After the lesson, the performance of the students increased statistically significantly. However, there was no significant difference between the groups in the shade‐matching performance research conducted by Haddad et al.^50^ between undergraduate students and dentists. The results of this study, contrary to the last research, showed that undergraduate students with less experience were weaker in shade matching success, especially in non‐polarized technique.

Current intraoral scanners can determine the color in any recorded area of the teeth and can make color impressions. In the present study, the maxillary central incisor's color was determined after scanning each subject and compared with the colors detected by Vita Easyshade. However, it was found that only one of the color determinations performed on 10 subjects was consistent with the results of Vita Easyshade. The consistency of 10% is characterized as poor, which corresponds to less than 20% on the strength of agreement scale. Yoon et al.,^41^ who measured the color determination performance of intraoral scanners on the shade tabs, reported that the results of the scanners and the colorimeter were not consistent, and in parallel with the present study, intraoral scanners were not reliable in terms of shade matching.

One of the limitations of this study is that the performance of the spectrophotometer can be affected by ambient lighting,^7^ which can change the data obtained on shade matching. In accordance with the inclusion criteria of the study, shade matching was carried out on young undergraduate students with intact teeth, but in clinical practice, shade matchings may be carried out on patients with more variable value levels, discolorations, and older restorations. This can affect both visual and instrumental shade matchings. In addition, there are several intraoral scanners in the market that offer color determination, and they can also be assessed in terms of shade‐matching performance. In the present study, only TRIOS 3 was used. In future studies, it will be useful to evaluate the performance of different shade‐matching techniques on subject groups with different age ranges and assess different up‐to‐date intraoral scanners.

## CONCLUSION

Within the limits of this in vivo study, shade matchings performed on digital dental photographs obtained with the cross‐polarization technique are not superior to the non‐polarization technique, and both techniques are not always clinically reliable. Clinical and academic experience may correlate, although not consistently, with shade‐matching success in digital photographs. Intraoral scanners are not sufficient for shade matching to be used in clinical practice.

## CONFLICT OF INTEREST STATEMENT

The authors declare no conflicts of interest.

## PREVIOUS PRESENTATION

Presented at the 25th ICP‐TPID Joint Meeting and 25th Scientific Congress of Turkish Prosthodontics and Implantology Association, Muğla, Turkey, November 2021.
